# Cognition of the association between sexual dysfunction and fertility

**DOI:** 10.1093/sexmed/qfaf084

**Published:** 2025-10-28

**Authors:** Yuying Sheng, Lewen Ruan, Yuge Chen, Xu Han, Baoyan Wu, Mingrong Lv, Dongdong Tang, Kuokuo Li

**Affiliations:** Reproductive Medicine Center, Department of Obstetrics and Gynecology, the First Affiliated Hospital of Anhui Medical University, Hefei 230022, China; NHC Key Laboratory of Study on Abnormal Gametes and Reproductive Tract, Anhui Medical University, Hefei 230032, Anhui, China; Key Laboratory of Population Health Across Life Cycle (Anhui Medical University), Ministry of Education of the People’s Republic of China, Hefei 230032 Anhui, China; Reproductive Medicine Center, Department of Obstetrics and Gynecology, the First Affiliated Hospital of Anhui Medical University, Hefei 230022, China; NHC Key Laboratory of Study on Abnormal Gametes and Reproductive Tract, Anhui Medical University, Hefei 230032, Anhui, China; Key Laboratory of Population Health Across Life Cycle (Anhui Medical University), Ministry of Education of the People’s Republic of China, Hefei 230032 Anhui, China; Reproductive Medicine Center, Department of Obstetrics and Gynecology, the First Affiliated Hospital of Anhui Medical University, Hefei 230022, China; NHC Key Laboratory of Study on Abnormal Gametes and Reproductive Tract, Anhui Medical University, Hefei 230032, Anhui, China; Key Laboratory of Population Health Across Life Cycle (Anhui Medical University), Ministry of Education of the People’s Republic of China, Hefei 230032 Anhui, China; Reproductive Medicine Center, Department of Obstetrics and Gynecology, the First Affiliated Hospital of Anhui Medical University, Hefei 230022, China; NHC Key Laboratory of Study on Abnormal Gametes and Reproductive Tract, Anhui Medical University, Hefei 230032, Anhui, China; Key Laboratory of Population Health Across Life Cycle (Anhui Medical University), Ministry of Education of the People’s Republic of China, Hefei 230032 Anhui, China; Reproductive Medicine Center, Department of Obstetrics and Gynecology, the First Affiliated Hospital of Anhui Medical University, Hefei 230022, China; NHC Key Laboratory of Study on Abnormal Gametes and Reproductive Tract, Anhui Medical University, Hefei 230032, Anhui, China; Key Laboratory of Population Health Across Life Cycle (Anhui Medical University), Ministry of Education of the People’s Republic of China, Hefei 230032 Anhui, China; Reproductive Medicine Center, Department of Obstetrics and Gynecology, the First Affiliated Hospital of Anhui Medical University, Hefei 230022, China; NHC Key Laboratory of Study on Abnormal Gametes and Reproductive Tract, Anhui Medical University, Hefei 230032, Anhui, China; Key Laboratory of Population Health Across Life Cycle (Anhui Medical University), Ministry of Education of the People’s Republic of China, Hefei 230032 Anhui, China; Reproductive Medicine Center, Department of Obstetrics and Gynecology, the First Affiliated Hospital of Anhui Medical University, Hefei 230022, China; NHC Key Laboratory of Study on Abnormal Gametes and Reproductive Tract, Anhui Medical University, Hefei 230032, Anhui, China; Key Laboratory of Population Health Across Life Cycle (Anhui Medical University), Ministry of Education of the People’s Republic of China, Hefei 230032 Anhui, China; Reproductive Medicine Center, Department of Obstetrics and Gynecology, the First Affiliated Hospital of Anhui Medical University, Hefei 230022, China; NHC Key Laboratory of Study on Abnormal Gametes and Reproductive Tract, Anhui Medical University, Hefei 230032, Anhui, China; Key Laboratory of Population Health Across Life Cycle (Anhui Medical University), Ministry of Education of the People’s Republic of China, Hefei 230032 Anhui, China

**Keywords:** reproductive function cognition, erectile dysfunction, premature ejaculation, sexual intercourse pain, sexual function

## Abstract

**Background:**

While research has shown that sexual dysfunction does not impact fertility, public understanding of this relationship remains unclear.

**Aim:**

To investigate the relationship between public cognition of the association between sexual dysfunction and fertility (ASDF) and the occurrence of sexual dysfunction itself.

**Methods:**

This cross-sectional study utilized a nationally representative sample of 10 761 reproductive-age individuals across China. Participants completed an anonymous questionnaire assessing sexual function and ASDF cognition, using tools like IIEF-5, PEDT, and FSFI-19. Data analysis was conducted with R software, employing χ^2^ and Mann–Whitney *U* tests.

**Outcomes:**

The study identified a significant correlation between ASDF cognition and the severity of sexual dysfunction, with lower levels of cognition associated with more severe dysfunction.

**Results:**

Among 10 761 surveyed, 45.08% of men and 54.92% of women showed low ASDF cognition. There was a significant correlation between ASDF cognition and sexual dysfunction severity, with poor cognition associated with more severe ED and PE in men, and sexual intercourse pain in women.

**Clinical Implications:**

The findings suggest the need for targeted sexual health education to improve public understanding of sexual dysfunction and its impact on fertility.

**Strengths and Limitations:**

Strengths include a large nationally representative sample and the use of validated tools. Limitations are the China-focused sample and subjective assessment tools, which may limit broader applicability.

**Conclusion:**

This study identifies a significant association between ASDF cognition and the severity of sexual dysfunction, underscoring the importance of public education on sexual health.

## Introduction

Sexual dysfunctions are characterized by disturbances in sexual desire and the psychophysiological changes associated with the sexual response cycle in both men and women.^1^ Previous studies indicate that sexual dysfunction is prevalent in both sexes, affecting approximately 10%-52% of men and 25%-63% of women.^2^ Sexual dysfunctions often affect the relationship and quality of life of couples. Among women, the most commonly reported issues are low sexual desire and difficulty achieving orgasm.^3^ Moreover, a significant proportion of women show difficulty with sexual arousal or sexual intercourse pain during sexual stimulation (dyspareunia), and particularly debilitating for menopausal women.^4^ Men’s sexual problems predominantly consist of premature ejaculation (PE), erectile dysfunction (ED), and hypoactive sexual desire disorder (HSDD).^5^ For instance, the Massachusetts Male Aging Study (MMAS) reported ED in 34.8% of men with an age range of 40-70 years. Additionally, a small proportion of men report problems with sexual intercourse or pain during intercourse.^6^

Multiple etiologies—including psychogenic, vascular, endocrine, and iatrogenic factors—are associated with sexual dysfunctions in both men and women.^7^ ED is strongly associated with age, comorbidities (eg, diabetes, hypertension), psychological disturbances (eg, depression, anxiety), and demographic factors (eg, education, race/ethnicity).^8^ In addition, 1 previous study showed that there is a correlation between depression and the total Female Sexual Function Index-6 (FSFI-6) score.^9^ Women with depressed mood had a 2-fold increased risk of sexual dysfunction compared with women with normal mood (Center for Epidemiologic Studies Depression 10-item Scale total score < 10). Furthermore, the study found that women with depression tend to have FSFI-6 scores that are approximately 20% lower than those of women without depression.^10^

Existing research suggests that sexual dysfunction may lead to infertility in certain cases,^11^ but it does not have a direct impact on fertility.^12^ Physiological conditions, such as ED and PE, may impede successful intercourse, but do not inherently affect the biological processes of conception. Similarly, HSDD may reduce sexual activity frequency, yet it does not directly impair reproductive health.^13^ “The psychological issues (e.g, anxiety and depression) often correlated to sexual dysfunction do not necessarily translate to reduced fertility”.^14^^,^^15^ Epidemiological studies have shown inconsistent associations between sexual dysfunction and fertility across different populations, age groups, and regions, suggesting that the relationship is not straightforward.^16^ Clinical observations also indicate that many patients with sexual dysfunction can still achieve pregnancy, further supporting the notion that sexual dysfunction does not directly impair fertility.^17^

Although sexual dysfunction does not directly impair fertility, some individuals mistakenly believe that any sexual problem inevitably signals infertility, and this misconception can itself heighten sexual distress. Research on the association between the cognition of ASDF (the association between sexual dysfunction and fertility) and sexual dysfunction is limited.^18^ Hence, we pose the hypothesis: Is there a significant association between cognitive aspects of ASDF and the incidence of sexual dysfunction, and if so, what is the impact of this relationship on perceptions of fertility and reproductive health? To address this hypothesis, our study aims to investigate this potential link by analyzing questionnaire data that assesses sexual dysfunction and its perceived effects on fertility.

## Materials and methods

### Participants

In the initial phase of our study, we employed a regional quota sampling strategy designed to mirror the national population distribution accurately. Utilizing data from the China Health Statistical Yearbook, we segmented the country into 3 primary regions: Eastern, Central, and Western. These regions were allocated sample sizes in a 4:3:3 ratio, respectively, reflecting their population distribution. To account for the Eastern region’s higher population density and urbanization, we adjusted the sample distribution ratio between the combined Central and Western regions and the Eastern region to approximately 6:4. This resulted in 5255, 3704, and 3198 participants from the Eastern, Central, and Western regions, respectively, closely aligning with the national demographic ratios.

In the subsequent phase, we applied gender-based quotas within each region to achieve a balanced representation of male and female participants. These quotas were informed by the latest national census data, ensuring our sample’s gender composition reflected that of the general population. Consequently, our final sample comprised 5259 males and 5502 females, maintaining an approximate 1:1 gender ratio.

Following the collection of questionnaires, we have clearly defined our inclusion criteria to encompass individuals aged 20-40 years who are within the reproductive age group. To maintain the quality and integrity of our data, we established specific exclusion criteria. These criteria excluded questionnaires that exhibited logical inconsistencies (810 cases), indicated systematic attention detection issues (531 cases), and those that were completed in an unreasonably extended period (55 cases).

Then, ethical approval for our study was obtained from the Institutional Review Board of the First Affiliated Hospital of Anhui Medical University (ID: PJ 2024-08-87), and informed consent was secured from all participants involved in the study.

### Questionnaires

Participants were surveyed using an anonymous questionnaire. To enhance participants’ comprehension of the questionnaire and improve data accuracy, uniformly trained andrologists were required to guide participants in completing the survey. The questionnaire collected general information on participants’ Sex, Age, Population, Body mass index (BMI), BMI grade, Education, Income, Constitution (referring to the physical fitness or health status of the participants), Underlying disease, Depression score, Depression grade, Age at first sexual intercourse, Frequency Sex. We estimated sexual function using the International Index of Erectile Function-5 (IIEF-5), Premature Ejaculation Diagnostic Tool (PEDT), and Female Sexual Function Index-19 (FSFI-19). The IIEF-5 is a commonly used tool to assess the severity of ED. The rating scale comprised 5 items: “Confidence in achieving and maintaining an erection,” “Frequency of erections,” “Ability to maintain an erection during intercourse,” “Difficulty in maintaining an erection,” and “Satisfaction with intercourse.” Each item was scored on a scale ranging from 1 to 5. The phenotype of ED is graded into 5 levels according to the score of IIEF-5, including severe (≤7), moderate (8-11), mild to moderate (12-16), mild (17-21), and no ED (22-25).^19^ PEDT is a tool designed to evaluate the presence of PE. Internationally, the evaluation of PE mainly focuses on the ability of men to control ejaculation, and time is a relatively minor criterion. The score of each item in the PEDT ranges from 0 to 4. The presence of PE is graded into 3 levels according to the total score of PEDT, including: PE (≥11), Probable PE (9-10), and No PE (≤8).^20^^,^^21^ The FSFI-19 is used to assess women’s sexual function across 6 separate domains: desire, arousal, lubrication, orgasm, satisfaction, and pain. Each domain includes a different number of questions, and each question is scored on a scale from 0 to 5. The phenotype of women’s sexual function is graded into 2 levels according to the total score of FSFI-19: Female sexual dysfunction (FSD) (≤26.55) and No FSD (>26.55).^22^ The total FSFI-19 score is the sum of all domain scores, with a higher total score indicating better overall sexual function.

**Figure 1:**
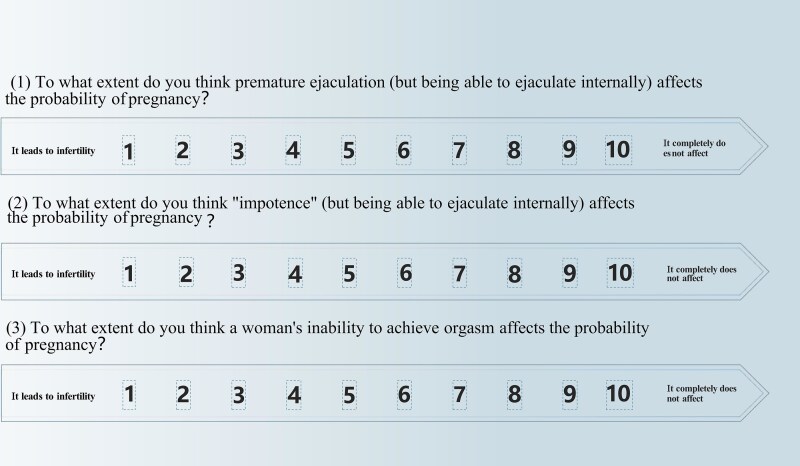
Cognitions of participants on the impact of male ED, male PE, and female orgasm on fertility. A questionnaire study: we have designed a questionnaire to survey men and women separately on their views about how sexual dysfunction affects fertility, in order to study the cognitive differences between the sexes, each problem scored on a scale of 0-10, with a passing score set at 6.

We designed 3 questions to estimate association between sexual dysfunction and cognition of ASDF: (1) To what extent do you think PE (but being able to ejaculate intravaginally) affects the probability of pregnancy? (2) To what extent do you think “impotence” (but being able to ejaculate intravaginally) affects the probability of pregnancy? (3) To what extent do you think a woman’s inability to achieve orgasm affects the probability of pregnancy? Each item was scored on a scale from 1 to 10, reflecting the extent to which participants perceived the impact of sexual dysfunction on fertility (Figure 1).^23^ A summary score of 18 was defined as the threshold for determining whether an individual demonstrated misunderstandings related to ASDF. Participants with scores < 18 were categorized as having low cognition of the impact of sexual dysfunction on fertility, whereas those with scores *n* 18 were categorized as having high cognition.

### Statistical analysis

Categorical variables are shown using descriptive statistics, including frequency counts and percentages. We used the χ^2^ test to compare categorical variables and the Mann–Whitney *U* test to analyze continuous variables, examining differences between the low- and high-cognition groups regarding the impact of sexual dysfunction on fertility. All statistical analyses were performed with R software (version 4.2.3). Statistical significance was defined as a bilateral *P* < .05.

## Results

### Sociodemographic characteristics of the participants

**Figure 2:**
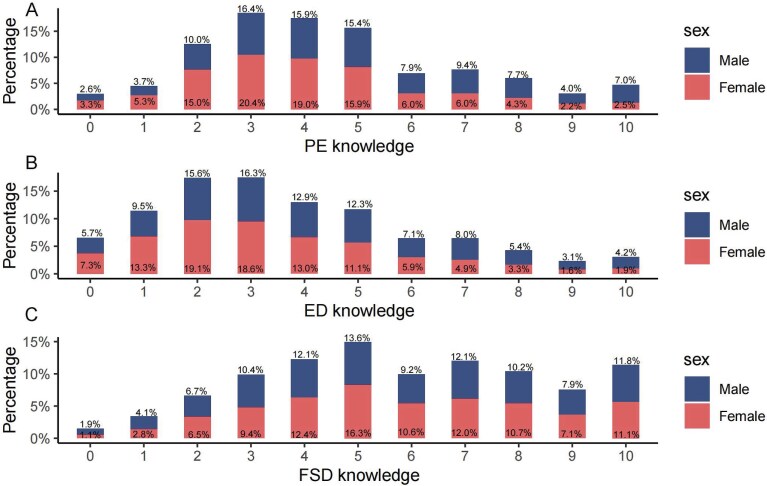
Analysis of cognitive gender differences in the impact of male ED, male PE, and female orgasm on fertility. The horizontal axis represents the categories of cognitive issues (PE knowledge, ED knowledge, FSD knowledge), and the vertical axis indicates the percentage of individuals of different sexes who have varying levels of understanding in each cognitive area. A: Bars indicate the proportion of men and women across different score levels on a PE knowledge assessment scale. B: Bars indicate the proportion of men and women across different score levels on an ED knowledge assessment scale. C: Bars indicate the proportion of men and women across different score levels on a FSD knowledge assessment scale.

A total of 13 465 questionnaires were distributed, and 12 157 were returned. Removing problems such as illogicality within the questionnaires and excessive time on answering the questions, we finally obtained 10 761 valid questionnaires with a median age of 29 years among the participants (IQR: 25.0-32.0). Additionally, 51.13% of the participants were women. In the questionnaire, cognition of ASDF was assessed through 3 questions addressing the perceived impact of PE, ED, and women’s inability to achieve orgasm on fertility (Figure 1). We found that men had higher cognition scores for PE (Figure 2A) and ED (Figure 2B) than women. Conversely, women had higher cognition scores for FSD (Figure 2C) than men. This finding suggests that both men and women tend to have a more comprehensive understanding of how their own sexual dysfunction affects fertility.

We divided the participants into high and low cognitive groups based on the threshold ASDF cognition score of 18.^24^ High cognition (ASDF cognition score ≥ 18) thought that sexual dysfunction had less impact on fertility, whereas low cognition (ASDF score < 18) thought that sexual dysfunction had more impact on fertility. There were 8012 participants with an ASDF cognition score < 18 (low cognition) and 2749 participants with a score ≥ 18 (high cognition) (Table 1). We found significant differences in in Sex (*P* < .01), Age (*P* < .01), BMI (*P* < .01), Education (*P* < .01), Income (*P* < .05), Constitution (*P* < .01), Underlying disease (*P* < .01), Depression score (*P* < .01), Age at first sex (*P* < .01), and Frequency of sex (*P* < .05) between individuals with low cognition and high cognition (Table 1). There was no statistical significance in Smoking, Drinking, Resident (“urban” or “rural”), Region, Personality (introverted or extroverted), and Pregnancy (total number of pregnancies) between individuals with low cognition and high cognition (Table S1).

**Table 1:** Descriptive characteristics of the study participants.

**Characteristics**	**Level**	**Total (*n* = 10 761)**	**<18 (*n* = 8012)**	**≥18 (*n* = 2749)**	***P* value**
Sex (%)	Male	5259 (48.87)	3612 (45.08)	1647 (59.91)	<.01
	Female	5502 (51.13)	4400 (54.92)	1102 (40.09)	
Age		29 [25,32]	28 [25, 32]	29 [25, 33]	<.01
Population (%)	Han	10 141 (94.24)	7526 (93.93)	2615 (95.13)	<.05
	Non-Han	620 (5.76)	486 (6.07)	134 (4.87)	
BMI		20.94 [19.15, 23.03]	20.81 [19.05, 22.86]	21.38 [19.53, 23.51]	<.01
BMI_grade (%)	Thin	1810 (16.82)	1446 (18.05)	364 (13.24)	<.01
	Normal	7147 (66.42)	5344 (66.70)	1803 (65.59)	
	Overweight	1804 (16.76)	1222 (15.25)	582 (21.17)	
Education (%)	High school or below	808 (7.51)	574 (7.16)	234 (8.51)	<.01
	Undergraduate	8848 (82.22)	6654 (83.05)	2194 (79.81)	
	Postgraduate or higher	1105 (10.27)	784 (9.79)	321 (11.68)	
Income (%)	<5 K ¥/m	2915 (27.09)	2209 (27.57)	706 (25.68)	<.05
	5 K-10 K ¥/m	5653 (52.53)	4221 (52.68)	1432 (52.09)	
	>10 K ¥/m	2193 (20.38)	1582 (19.75)	611 (22.23)	
Constitution (%)	Poor	630 (5.85)	496 (6.19)	134 (4.87)	<.01
	Moderate	6603 (61.36)	4983 (62.19)	1620 (58.93)	
	Good	3528 (32.79)	2533 (31.62)	995 (36.19)	
Underlying disease (%)	None	8594 (79.86)	6287 (78.47)	2307 (83.92)	<.01
	Yes	2167 (20.14)	1725 (21.53)	442 (16.08)	
Depression score		6.00 [4.00, 10.00]	6.00 [4.00, 10.00]	5.00 [3.00, 8.00]	<.01
Depression grade (%)	None	3736 (34.72)	2629 (32.81)	1107 (40.27)	<.01
	Light	6168 (57.32)	4686 (58.49)	1482 (53.91)	
	Medium	857 (7.96)	697 (8.70)	160 (5.82)	
Age at first sex		22 [20, 24]	22 [20, 24]	22 [20, 24]	<.01
Frequency sex (%)	<1	3678 (34.18)	2755 (34.39)	923 (33.58)	<.05
	1-2	5003 (46.49)	3751 (46.82)	1252 (45.54)	
	3-5	1863 (17.31)	1361 (16.99)	502 (18.26)	
	≥0	217 (2.02)	145 (1.81)	72 (2.62)	

Value in square brackets represent the median, and value in parentheses represent percentage. BMI, body mass index; Income <5000 ¥/m is monthly income. We used the χ^2^ test for compare categorical variables, and the Mann–Whitney *U* test for continuous variables. Statistical significance was defined as a bilateral *P* < .05.

### Cognitive association with men sexual dysfunction

We found a high proportion of man respondents with low ASDF cognition (3612/5259, 68.68%) (Table 1). Therefore, we focused on exploring the association between ASDF cognition with man sexual function, including ED and PE, by IIEF-5 and PEDT (Table 2). Compared to men with ASDF cognition score ≥ 18 (IIEF-5 median: 21, IQR: 19.0-23.0), those with ASDF cognition score < 18 (IIEF-5 median: 20, IQR: 17.0-23.0) had lower IIEF-5 score, indicating a more severe ED (*P* < .01). To further illustrate the relationship between cognition and ED, we classified ED into 5 categories based on IIEF-5 scores: severe (≤7), moderate (8-11), mild to moderate (12-16), mild (17-21), and no ED (22-25). Specifically, the prevalence of ED was higher in men with ASDF score < 18 (1-37.24%, 62.76%) compared to those with ASDF score ≥ 18 (1-48.45%, 51.55%). For the assessment of PE, men with ASDF score < 18 had higher PEDT score (median: 11, IQR: 8-13) than those with ASDF score ≥ 18 (median: 10, IQR: 8-12), indicating a more severe PE (*P* < .01). To further examine the relationship between ASDF cognition and PE, we categorized PEDT scores into 3 groups: “PE” (≥11), “probable PE” (9-10), and “no PE” (≤8). The prevalence of PE was higher among men with an ASDF cognition score < 18 (51.08%) than among those with a score ≥ 18 (23.19%)^21^ (Table 2). To further analyze the correlation between ASDF cognition and man sexual dysfunction, we employed univariate linear regression analysis to precisely assess the specific trends and also found similar results that ASDF cognition was correlated negatively with ED (IIEF-5: β = 0.08, *P* < .05) and PE (PEDT: β = −0.06, *P* < .01) (Table 3).

**Table 2:** The impact of male cognitive scores on sexual dysfunction on sexual function.

**Characteristics**	**Level**	**Total (*N* = 5259)**	**<18 (*n* = 3612)**	**≥18 (*n* = 1647)**	***P* value**
IIEF5 score		21 [18, 23]	20 [17, 23]	21 [19, 23]	<.01
IIEF5 grade (%)	Severe	270 (5.13)	170 (4.71)	100 (6.07)	<.01
	Moderate	113 (2.15)	97 (2.69)	16 (0.97)	
	Mild to moderate	636 (12.09)	514 (14.23)	122 (7.41)	
	Mild	2097 (39.87)	1486 (41.14)	611 (37.10)	
	Normal	2143 (40.75)	1345 (37.24)	798 (48.45)	
PEDT score		10 [8, 13]	11 [8, 13]	10 [8, 12]	<.01
PEDT grade (%)	PE probable PE	2227 (42.35) 1463 (27.82)	1845 (51.08) 753 (20.85)	382 (23.19) 710 (43.11)	<.01
	No PE	1569 (29.83)	1014 (28.07)	555 (33.70)	
PEDT fertility		5 [3, 7]	4 [3, 5]	8 [7, 9]	<.01
IIEF5 fertility		4 [2, 6]	3 [2, 4]	7 [5, 8]	<.01
PEDT fertility grade (%)	Impact	3368 (64.04)	3169 (87.74)	199 (12.08)	<.01
	No impact	1891 (35.96)	443 (12.26)	1448 (87.92)	
IIEF5 fertility grade (%)	Impact	3803 (72.31)	3370 (93.30)	433 (26.29)	<.01
	No impact	1456 (27.69)	242 (6.70)	1214 (73.71)	

Value in square brackets represent the median, and value in parentheses represent percentage. IIEF-5, International Index of Erectile Function-5. ED, erectile dysfunction. IIEF5 grade: IIEF5 scores ≤7 for Severe ED; IIEF5 scores: 8-11 for Moderate ED; IIEF5 scores: 12-16 for Mild to Moderate ED; IIEF5 scores: 17-21 for Mild ED; IIEF5 scores: 22-25 for No ED. PEDT, Premature Ejaculation Diagnostic Tool. PE, Premature Ejaculation. PEDT grade: PE (PEDT scores ≥10), No PE (PEDT scores ≤9). PEDT fertility: refers to the perception of whether or not PE affects fertility. IIEF5 fertility: refers to the perception of whether or not ED affects fertility.

**Table 3:** Relationship between cognitive scores on sexual dysfunction and male sexual function.

**Variable**	**SD fertility (univariate linear regression model)**		**SD fertility (multivariate linear regression model)**			
	**β**	**95% CI**	***P* value**	**β**	**95% CI**	***P* value**
PEDT score	−0.06	−0.08 to −0.04	<.01	−0.02	−0.03 to −0.004	<.05
IIEF5 score	0.08	0.06 to 0.09	<.05	0.04	0.03 to 0.06	<.01

We initially constructed univariate linear regression models to analyze the relationships among SD fertility, PEDT score, and IIEF5 score. Then, we controlled for factors such as depression score, population, smoking, drinking, age, BMI grade, education, income, pregnancy, resident, region, personality, constitution, underlying disease, and fertility desire by employing multivariate linear regression models. These models were utilized to further investigate the impact of SD fertility on PEDT score and IIEF5 score.

Moreover, given the potentially multifactorial nature of sexual function, we further explored the association of other factors with sexual function. Depression score (OR = 0.87, *P* < .01), Education (OR = 1.42, *P* < 0.05), Income (OR = 2.58, *P* < .01), Personality (OR = 1.33, *P* < .05), and Constitution (OR = 3.27, *P* < 0.01) show a significant correlation with IIEF-5 score (Table S2). We also found that Depression score (OR = 1.24, *P* < .01), Personality (OR = 0.67, *P* < .01), and Constitution (OR = 0.54, *P* < .01) were significantly correlated with PEDT score (Table S2). Men with less depression, better education, income, extroversion, and health typically suggested lower sexual dysfunction risk (Table S2). We controlled for factors such as Depression score, Population, Smoking, Drinking, Age, BMI grade, Education, Income, Pregnancy, Resident, Region, Personality, Constitution, Underlying disease, and Fertility desire by employing multivariate linear regression models. We observed similar findings: ASDF cognition significantly affects ED (β = 0.04, *P* < .01) and PE (β = −0.02, *P* < .05) in men of reproductive age (Table 4) (Figure S1).

**Table 4:** The impact of female cognitive scores on sexual dysfunction on sexual function.

**Characteristics**	**Level**	**Total (*N* = 5502)**	**<18 (*n* = 4400)**	**≥18 (*n* = 1102)**	***P* value**
Desire score of FSFI-19		3.6 [2.4, 4.2]	3.6 [2.4, 4.2]	3.6 [2.4, 4.2]	.68
Arousal score of FSFI-19		3.6 [2.7, 4.5]	3.6 [2.7, 4.5]	3.6 [2.7, 4.5]	.05
Lubrication score of FSFI-19		4.5 [3.6, 5.4]	4.5 [3.6, 5.4]	4.8 [3.9, 5.7]	<.01
Orgasm score of FSFI-19		4 [2.8, 4.8]	4 [3.2, 4.8]	4 [2.8, 4.8]	.78
Satisfaction score of FSFI-19		4.8 [3.6, 5.2]	4.4 [3.6, 5.2]	4.8 [3.6, 5.2]	0.07
Pain score of FSFI-19		4.4 [3.2, 5.2]	4.4 [3.2, 4.8]	4.8 [3.2, 5.6]	<.01
FSFI-19 total score		24.5 [19.9, 28.1]	24.4 [19.9, 27.9]	24.9 [20.1, 28.88]	<.01
FSFI-19 total score grade (%)	No FSD	1966 (35.73)	1519 (34.52)	447 (40.56)	<.01
	With FSD	3536 (64.27)	2881 (65.48)	655 (59.44)	
Female orgasm fertility		6 [4, 8]	5 [4, 7]	9 [8, 10]	<.01
SD fertility		13 [9, 16]	11 [9, 14]	22 [19, 25]	<.01
Female orgasm fertility grade (%)	Impact	2670 (48.53)	2619 (59.52)	51 (4.63)	<.01
	No impact	2832 (51.47)	1781 (40.48)	1051 (95.37)	

OR refers to “Odds Ratio”, 95% CI refers to “95% Confidence Interval”. Square brackets represent the median, and parentheses represent *n* (%). FSFI-19 is a scale for assessing female sexual function, and FSD refers to female sexual dysfunction. FSFI-19 total score grade: FSFI-19 scores >26.55 for No FSD; FSFI-19 scores ≤26.55 for with FSD. Female orgasm fertility: refers to the perception of whether or not to achieve orgasm affects fertility. SD fertility is the total score of 3 cognitive questions we designed. Female orgasm fertility grade: female orgasm fertility scores ≥6: Impact; female orgasm fertility scores <6: No Impact.

### Cognitive association with women sexual dysfunction

We also found a high proportion of women respondents with low ASDF (4400/5502, 79.97%). Therefore, we focused on exploring the association between ASDF with women sexual function by the FSFI-19. Women with ASDF score ≥ 518 exhibited higher FSFI-19 total score (median: 24.9, IQR: 20.1-28.9, *P* < .01), Lubrication score of FSFI-19 (median: 4.8, IQR: 3.9-5.7, *P* < .01), and Pain score of FSFI-19 (median: 4.8, IQR:3.2-5.6, *P* < .01) than those with ASDF score < 18, which indicated that lower ASDF cognition score are associated with more severe FSD (Table 3). We categorized the FSFI-19 total score into 2 groups: FSD (≤26.55) and no FSD (>26.55), and found that the prevalence of FSD was higher in women with ASDF cognition score < 18 (65.48%) compared to those with ASDF cognition score e 18 (59.44%) (Table 4).

Meanwhile, we also further explored the association of other factors with sexual dysfunction. These factors included the Depression score (OR = 0.84, *P* < .01), BMI (OR = 0.88, *P* < .01), Income (OR = 5.08, *P* < .01), Residence (OR = 0.24, *P* < .01), Personality (OR = 4.97, *P* < .01), Constitution (OR = 6.03, *P* < .01), and Fertility desire (OR = 13.12, *P* < .01). Women who are mentally positive, have healthy BMI values, high incomes, live in urban areas, are extroverted, and have good physical constitutions may face fewer risks of sexual dysfunction (Table S3).

We adjusted for factors such as Depression score, Population, Smoking, Drinking, Age, BMI grade, Education, Income, Pregnancy, Resident, Region, Personality, Constitution, Underlying disease, Fertility desire by employing multivariate linear regression models, we found that the significant effects on lubrication (β = 0.008, *P* < .05) and pain (β = 0.01, *P* < .01) observed in univariate linear regression models were no longer significant for lubrication (β = 0.005, *P* = .14). Furthermore, ASDF cognition was found to be unrelated to FSFI-19 total score (β = −0.012, *P* = 0.47), desire (β = −0.004, *P* = .08), arousal (β = −0.006, *P* = .06), orgasm (β = −0.013, *P* < .01), and satisfaction (β = −0.004, *P* = .30) consistently. This suggests that a higher ASDF cognition score is associated with reduced pain during sexual activity (Table 5) (Figure S1).

**Table 5:** Relationship between cognitive scores on sexual dysfunction and sexual function.

**Variable**	**SD fertility (univariate linear regression model)**			**SD fertility (multivariate linear regression model)**		
	**β**	**95% CI**	***P* value**	**β**	**95% CI**	***P* value**
Desire score of FSFI-19	−0.006	−0.01 to −0.001	<.05	−0.004	−0.009 to 0.0005	.08
Arousal score of FSFI-19	−0.006	−0.01 to 0.001	.10	−0.006	−0.01 to 0.0003	.06
Lubrication score of FSFI-19	0.008	0.001 to 0.02	.04	0.005	−0.002 to 0.01	.14
Orgasm score of FSFI-19	−0.01	−0.01 to −0.003	<.01	−0.01	−0.02 to −0.006	<.01
Satisfaction score of FSFI-19	−0.002	−0.001 to 0.006	.68	−0.004	−0.01 to 0.003	.30
Pain score of FSFI-19	0.01	0.006 to 0.02	<.01	0.009	0.002 to 0.01	<.05
FSFI-19 total score	−0.002	−0.04 to 0.04	.93	−0.01	−0.05 to 0.02	.47

We initially constructed univariate linear regression models to analyze the relationships among SD fertility, Desire score of FSFI-19, Arousal score of FSFI-19, Lubrication score of FSFI-19, Orgasm score of FSFI-19, Satisfaction score of FSFI-19, and Pain score of FSFI-19. Then, we controlled for factors such as depression score, population, smoking, drinking, age, BMI grade, education, income, pregnancy, resident, region, personality, constitution, underlying disease, and fertility desire by employing multivariate linear regression models. These models were utilized to further investigate the impact of SD fertility on Desire score of FSFI-19, Arousal score of FSFI-19, Lubrication score of FSFI-19, Orgasm score of FSFI-19, Satisfaction score of FSFI-19, and Pain score of FSFI-19.

### Sample clustering

To elucidate the latent traits and behavioral patterns inherent to distinct demographic groups, we selected a total of 15 variables to analyze the population (Table S4). These variables include demographic factors such as Age, Sex, Education, Income, Smoking, Drinking, Personality, and Constitution. Due to the inclusion of both continuous variables (eg, age, income) and categorical variables (eg, sex, smoking status), we decided to use the K-Prototypes algorithm to cluster the subject characteristics data. Using the Elbow method, a technique for determining the optimal number of clusters, we classified participants into 3 distinct clusters: cluster 1 (*n* = 1311), cluster 2 (*n* = 2184), and cluster 3 (*n* = 1552) (Table S4). We observed notable differences among the 3 clusters. For instance, cluster 2 exhibited a higher proportion of current smokers (67.4%) (*P* < .01) and drinkers (75.6%) (*P* < .01) a higher prevalence of extroverted individuals (84.0%) (*P* < .01), and a greater number of subjects who had experienced having a singleton child (50.6%) (*P* < .01) compared to clusters 1 and 3 (Table S4). This suggests that these demographic characteristics may be further explored as potential factors influencing reproductive function.

## Discussion

Sexual dysfunction may lead to inappropriate sexual behaviors.^25^ However, it does not have a direct impact on fertility.^26^ Several etiologies, including age, health conditions, emotion, and demographics, are associated with specific sexual dysfunctions.^27^ However, current societal understanding of the relationship between sexual dysfunction and fertility is generally lacking in population data. In the present study, we integrated a questionnaire from a nationally representative sample from the central, western, and eastern regions of China and systematically evaluated sexual dysfunction and ASDF cognition. We found that low ASDF cognition was associated with sexual dysfunction in both men (ED, PE) and women (sexual intercourse pain).

Studies show that men with prostate cancer^28^ or ED^29^ often maintain optimism and take proactive actions, such as seeking medical help. This may be influenced by societal expectations of being viewed as strong and autonomous. We focused on exploring how the ASDF cognition affects ED and PE in men of reproductive age. Our key result indicates that a diminished ASDF cognition score is correlated with the severity of ED and PE. This finding is consistent with the studies of Ayribas and Toprak (2021) and Slayday et al. (2023),^30^^,^^31^ who also pointed out the significant correlation between cognitive level and sexual function. In particular, Ayribas and Toprak (2021) found that patients with PE may have social cognitive deficits, characterized by an anxious attachment style that does not shy away from intimacy. Slayday et al. (2023) suggested that a decline in sexual health may be associated with an increased risk of cognitive ability decline. This may be related to the influence of cognition on behavioral patterns, which can indirectly affect cardiovascular health and, in turn, sexual function.^32^ In terms of FSD, we found that a lower ASDF score is significantly related to more severe sexual intercourse pain. This is consistent with the research results of Lamba et al. (2023), who found that a negative cognitive level is associated with an increase in sexual intercourse pain and vaginismus, which are strongly related to certain religious traditional concepts, such as remaining a virgin.^33^

Our analysis indicated that cluster 2, characterized by smokers, drinkers, and extroverted individuals, had a higher incidence of having a single child. This trend may relate to lifestyle factors that influence family planning, such as delayed parenting due to active social engagement common among extroverts.^34^ The unhealthy habits of smoking and drinking, prevalent in this group, can adversely affect fertility, with smoking known to impair blood flow essential for erection and conception.^35^^,^^36^ These factors may indirectly decrease the likelihood of having multiple children. Given the influence of lifestyle on sexual health, tailored sexual health education and family planning strategies are warranted for individuals with such characteristics. Additionally, cognitive therapy can be employed, focusing on helping individuals recognize and modify detrimental thought patterns and beliefs. This process aims to foster constructive emotional and behavioral changes, which may encourage healthier lifestyle choices and enhance sexual well-being, ultimately informing more considered family planning decisions.^37–39^

Our study distinguishes itself from the existing body of literature through its robust sample size and innovative methodology, providing a unique perspective on the intricacies of sexual dysfunction. We used a large-scale sample and designed a targeted questionnaire, which more clearly demonstrates the relationship between ASDF cognition and sexual dysfunction.

Meanwhile, we acknowledge several limitations of our study. First, the sample was drawn primarily from China, which may limit the generalizability of our findings to populations in other countries. Second, although sexual function assessments are widely used measurement tools, they remain subject to individual variability and subjective bias. We must exercise caution when interpreting our findings and ensure that they are considered alongside other pertinent research. This approach will allow for a more holistic and nuanced evaluation of sexual function issues.

Then, several limitations regarding the assessment tools and questionnaire design should be explicitly acknowledged. Firstly, while the IIEF-5 is widely accepted, it may not be as accurate in a phenotype assessing individuals with co-occurring ED and PE as the IIEF-EF domain.^40^ This highlights a potential underrepresentation of the complexity of sexual dysfunction within our assessment. Specifically, our questionnaire, despite being extensive, did not cover critical aspects such as the inability to achieve vaginal penetration due to ED, anejaculation, and unconsummated marriages. This omission may impact the comprehensiveness of our findings regarding sexual dysfunction. Additionally, although our study primarily focused on the cognitive impact of sexual dysfunction, our questionnaire design included a broad category for “underlying diseases” rather than detailed information on specific comorbidities. While this approach captured a wide range of potential health conditions, it limited our ability to discern the differential impact of various diseases on sexual function and/or infertility. We have acknowledged these limitations in our analysis and discussion, and we suggest that future studies could benefit from a more inclusive set of questions and a more detailed assessment of comorbidities to provide a more thorough evaluation of sexual dysfunction and a richer understanding of the complex relationships between underlying diseases and sexual function.

In summary, the findings suggest that a proportion of Chinese men and women have sexual dysfunction, which may be related to the cognition that sexual function affects reproductive function.

## Conclusion

Our research underscores the importance of enhancing public awareness and education regarding sexual health to improve cognitive understanding of the ASDF.

## Supplementary Material

Supplementary_File_qfaf084
