# Supplementary material for: Cognition of the association between sexual dysfunction and fertility
Source: Sex Med. 2025 Oct 28;13(5):qfaf084. doi: 10.1093/sexmed/qfaf084 (PMC12560785; doi:10.1093/sexmed/qfaf084)
Supplement: Supplementary_File_qfaf084 [file supplementary_file_qfaf084.docx]

**Methodology**

In the initial phase of our study, we employed a regional quota sampling strategy designed to mirror the national population distribution accurately. Utilizing data from the China Health Statistical Yearbook, we segmented the country into three primary regions: Eastern, Central, and Western. These regions were allocated sample sizes in a 4:3:3 ratio, respectively, reflecting their population distribution. To account for the Eastern region's higher population density and urbanization, we adjusted the sample distribution ratio between the combined Central and Western regions and the Eastern region to approximately 6:4. This resulted in 5,255, 3,704, and 3,198 participants from the Eastern, Central, and Western regions, respectively, closely aligning with the national demographic ratios.

In the subsequent phase, we applied gender-based quotas within each region to achieve a balanced representation of male and female participants. These quotas were informed by the latest national census data, ensuring our sample's gender composition reflected that of the general population. Consequently, our final sample comprised 5,259 males and 5,502 females, maintaining an approximate 1:1 gender ratio.

Following the collection of questionnaires, we have clearly defined our inclusion criteria to encompass individuals aged 20 to 40 years who are within the reproductive age group. To maintain the quality and integrity of our data, we established specific exclusion criteria. These criteria excluded questionnaires that exhibited logical inconsistencies (810 cases), indicated systematic attention detection issues (531 cases), and those that were completed in an unreasonably extended period (55 cases).

Then, ethical approval for our study was obtained from the Institutional Review Board of the First Affiliated Hospital of Anhui Medical University (ID: PJ 2024-08-87), and informed consent was secured from all participants involved in the study.

**Table S1.** **Descriptive characteristics of the study participants**

| **Characteristics** | **Level** | **Total (N = 10761)** | **<18 (n = 8012)** | **≥18 (n = 2749)** | **P value** |
| --- | --- | --- | --- | --- | --- |
| Smoking (%) | Never | 7384 (68.62) | 5527 (68.98) | 1857 (67.55) | 0.06 |
|  | Abstained | 989 (9.19) | 750 (9.36) | 239 (8.69) |  |
|  | Currently smoking | 2388 (22.19) | 1735 (21.66) | 653 (23.75) |  |
| Drinking (%) | Never | 5467 (50.80) | 4098 (51.15) | 1369 (49.80) | 0.32 |
|  | Abstained | 869 (8.08) | 653 (8.15) | 216 (7.86) |  |
|  | Currently drinking | 4425 (41.12) | 3261 (40.70) | 1164 (42.34) |  |
| Resident (%) | Urban | 9819 (91.25) | 7306 (91.19) | 2513 (91.42) | 0.75 |
|  | Rural | 942 (8.75) | 706 (8.81) | 236 (8.58) |  |
| Region (%) | Eastern | 4674 (43.43) | 3473 (43.35) | 1201 (43.69) | 0.25 |
|  | Central | 3249 (30.19) | 2395 (29.89) | 854 (31.07) |  |
|  | Western | 2838 (26.37) | 2144 (26.76) | 694 (25.25) |  |
| Personality (%) | Introversion | 5341 (49.63) | 3959 (49.41) | 1382 (50.27) | 0.45 |
|  | Extroversion | 5420 (50.37) | 4053 (50.59) | 1367 (49.73) |  |
| pregnancy (%) | 0 | 4300 (39.96) | 3220 (40.19) | 1080 (39.29) | 0.62 |
|  | 1 | 4114 (38.23) | 3063 (38.23) | 1051 (38.23) |  |
|  | 2 | 1833 (17.03) | 1357 (16.94) | 476 (17.32) |  |
|  | 3 | 514 (4.78) | 372 (4.64) | 142 (5.17) |  |

Note: Square brackets represent the median, and parentheses represent the n (%).

**Table S2. Risk factors affecting the male sexual dysfunction**

| **Variable** | **PEDT score** | | | **IIEF5 score** | | |
| --- | --- | --- | --- | --- | --- | --- |
|  | **OR** | **95%CI** | **P value** | **OR** | **95%CI** | **P value** |
| SD fertility | 0.97 | 0.95 - 0.98 | 3.45E-06 | 1.02 | 1.01 - 1.04 | < 0.05 |
| Depression score | 1.24 | 1.22 - 1.27 | < 2E-16 | 0.87 | 0.84 - 0.90 | < 0.01 |
| IIEF5 score/  PEDT score | 0.88 | 0.86 - 0.90 | < 2E-16 | 0.80 | 0.77 - 0.83 | < 0.01 |
| Age | 1.08 | 1.06 - 1.10 | 1.52E-10 | 1.08 | 1.05 - 1.11 | < 0.01 |
| BMI | 1.00 | 0.97 - 1.04 | 9.10E-01 | 0.97 | 9.21 - 1.00 | 0.07 |
| Education | 0.94 | 0.76 - 1.17 | 6.00E-01 | 1.42 | 1.07 - 1.89 | < 0.05 |
| Income | 1.15 | 0.97 - 1.35 | 9.80E-02 | 2.58 | 2.09 - 3.20 | < 0.01 |
| Pregnancy | 1.26 | 1.10 - 1.44 | 6.50E-04 | 1.80 | 1.51 - 2.14 | < 0.01 |
| Resident | 1.03 | 0.73 - 1.46 | 8.50E-01 | 0.67 | 0.43 - 1.05 | 0.08 |
| Region | 0.96 | 0.85 - 1.07 | 4.40E-01 | 0.87 | 0.75 - 1.01 | 0.06 |
| Personality | 0.67 | 0.55 - 0.81 | 6.69E-05 | 1.33 | 1.02 - 1.73 | < 0.05 |
| Constitution | 0.54 | 0.45 - 0.65 | 7.99E-11 | 3.27 | 2.57 - 4.17 | < 0.01 |
| Underlying disease | 1.94 | 1.52 - 2.46 | 7.85E-08 | 0.47 | 0.34 - 0.64 | < 0.01 |
| Fertility desire | 1.80 | 1.50 - 2.17 | 5.47E-10 | 2.08 | 1.63 - 2.67 | < 0.01 |

Note: OR refers to "Odds Ratio", 95%CI refers to "95% Confidence Interval". SD fertility is the total score of three cognitive questions. Premature Ejaculation Diagnostic Tool (PEDT) and International Index of Erectile Function-5 (IIEF-5) are scales for assessing male PE and ED; ED: erectile dysfunction, PE: Premature Ejaculation. BMI: body mass index.

**Table S3. Risk factors affecting the female sexual dysfunction**

| **Variables** | **FSFI - 19 score** | | |
| --- | --- | --- | --- |
|  | **OR** | **95%CI** | **P value** |
| SD fertility | 0.99 | 0.95 - 1.02 | 0.45 |
| Depression score | 0.84 | 0.80 - 0.88 | < 0.01 |
| Age | 1.02 | 0.97 - 1.08 | 0.37 |
| BMI | 0.88 | 0.82 - 0.94 | < 0.01 |
| Education | 0.45 | 0.27 - 0.76 | < 0.01 |
| Income | 5.08 | 3.63 - 7.12 | < 0.01 |
| Pregnancy | 5.69 | 4.34 - 7.47 | < 0.01 |
| Resident | 0.24 | 0.12 - 0.48 | < 0.01 |
| Region | 1.19 | 0.93 - 1.52 | 0.16 |
| Personality | 4.97 | 3.31 - 7.45 | < 0.01 |
| Constitution | 6.03 | 4.13 - 8.83 | < 0.01 |
| Underlying disease | 1.14 | 0.67 - 1.93 | 0.63 |
| Fertility desire | 13.12 | 8.86 - 19.43 | < 0.01 |

Note: OR refers to "Odds Ratio", 95%CI refers to "95% Confidence Interval". SD fertility is the total score of three cognitive questions we designed; Female Sexual Function Index-19 (FSFI-19) is a scale for assessing female sexual function. BMI: body mass index.

**Table S4. Demographic data for three clusters (n = 5047)**

| **Variables** | **Level** | **Cluster 1 (n = 1311)** | **Cluster 2 (n = 2184)** | **Cluster 3 (n = 1552)** | **P value** |
| --- | --- | --- | --- | --- | --- |
| age |  | 23.00 [22.00, 26.00] | 30.00 [28.00, 33.00] | 32.00 [28.00, 35.00] | < 0.05 |
| BMI grade (%) | Thin | 180 (13.7) | 121 ( 5.5) | 91 ( 5.9) | < 0.05 |
|  | Normal | 868 (66.2) | 1548 (70.9) | 986 (63.5) |  |
|  | Overweight | 263 (20.1) | 515 (23.6) | 475 (30.6) |  |
| smoking (%) | Never | 922 (70.3) | 378 (17.3) | 1031 (66.4) | < 0.05 |
|  | Abstained | 147 (11.2) | 333 (15.2) | 244 (15.7) |  |
|  | Currently smoking | 242 (18.5) | 1473 (67.4) | 277 (17.8) |  |
| drinking (%) | Never | 794 (60.6) | 303 (13.9) | 473 (30.5) | < 0.05 |
|  | Abstained | 120 ( 9.2) | 229 (10.5) | 174 (11.2) |  |
|  | Currently drinking | 397 (30.3) | 1652 (75.6) | 905 (58.3) |  |
| population (%) | Han | 1199 (91.5) | 2062 (94.4) | 1503 (96.8) | < 0.05 |
|  | Non-Han | 112 ( 8.5) | 122 ( 5.6) | 49 ( 3.2) |  |
| education (%) | High school or below | 131 (10.0) | 192 ( 8.8) | 125 ( 8.1) | ≥ 0.05 |
|  | Undergraduate | 1045 (79.7) | 1744 (79.9) | 1242 (80.0) |  |
|  | Postgraduate or Higher | 135 (10.3) | 248 (11.4) | 185 (11.9) |  |
| income (%) | < 5K /m | 768 (58.6) | 118 ( 5.4) | 127 ( 8.2) | < 0.05 |
|  | 5K-10K /m | 412 (31.4) | 1363 (62.4) | 982 (63.3) |  |
|  | > 10K /m | 131 (10.0) | 703 (32.2) | 443 (28.5) |  |
| resident (%) | Urban | 1114 (85.0) | 2067 (94.6) | 1461 (94.1) | < 0.05 |
|  | Rural | 197 (15.0) | 117 ( 5.4) | 91 ( 5.9) |  |
| region (%) | Eastern | 334 (25.5) | 974 (44.6) | 823 (53.0) | < 0.05 |
|  | Central | 431 (32.9) | 662 (30.3) | 406 (26.2) |  |
|  | Western | 546 (41.6) | 548 (25.1) | 323 (20.8) |  |
| personality (%) | Introversion | 888 (67.7) | 350 (16.0) | 1125 (72.5) | < 0.05 |
|  | Extroversion | 423 (32.3) | 1834 (84.0) | 427 (27.5) |  |
| Constitution (%) | Poor | 102 ( 7.8) | 64 ( 2.9) | 85 ( 5.5) | < 0.05 |
|  | Moderate | 848 (64.7) | 1182 (54.1) | 959 (61.8) |  |
|  | Good | 361 (27.5) | 938 (42.9) | 508 (32.7) |  |
| Underlying disease (%) | None | 1125 (85.8) | 1577 (72.2) | 1222 (78.7) | < 0.05 |
|  | Yes | 186 (14.2) | 607 (27.8) | 330 (21.3) |  |
| Depression score |  | 6.00 [4.00, 10.00] | 5.00 [4.00, 9.00] | 5.00 [3.00, 9.00] | < 0.05 |
| pregnancy (%) | 0 | 1176 (89.7) | 464 (21.2) | 359 (23.1) | < 0.05 |
|  | 1 | 81 ( 6.2) | 1106 (50.6) | 737 (47.5) |  |
|  | 2 | 44 ( 3.4) | 503 (23.0) | 362 (23.3) |  |
|  | 3 | 10 ( 0.8) | 111 ( 5.1) | 94 ( 6.1) |  |
| Fertility desire (%) | Impact | 629 (48.0) | 569 (26.1) | 1256 (80.9) | < 0.05 |
|  | No impact | 682 (52.0) | 1615 (73.9) | 296 (19.1) |  |
| IIEF5 score |  | 20.00 [17.00, 23.00] | 21.00 [19.00, 23.00] | 21.00 [19.00, 23.00] | < 0.05 |
| PEDT score |  | 10.00 [8.00, 13.00] | 10.00 [8.00, 13.00] | 10.00 [8.00, 13.00] | ≥ 0.05 |

Note: Square brackets represent the median, and parentheses represent n (%); Premature Ejaculation Diagnostic Tool (PEDT) and International Index of Erectile Function-5 (IIEF-5) are scales for assessing male PE and ED; Female Sexual Function Index-19 (FSFI-19) is a scale for assessing female sexual function; BMI: body mass index.


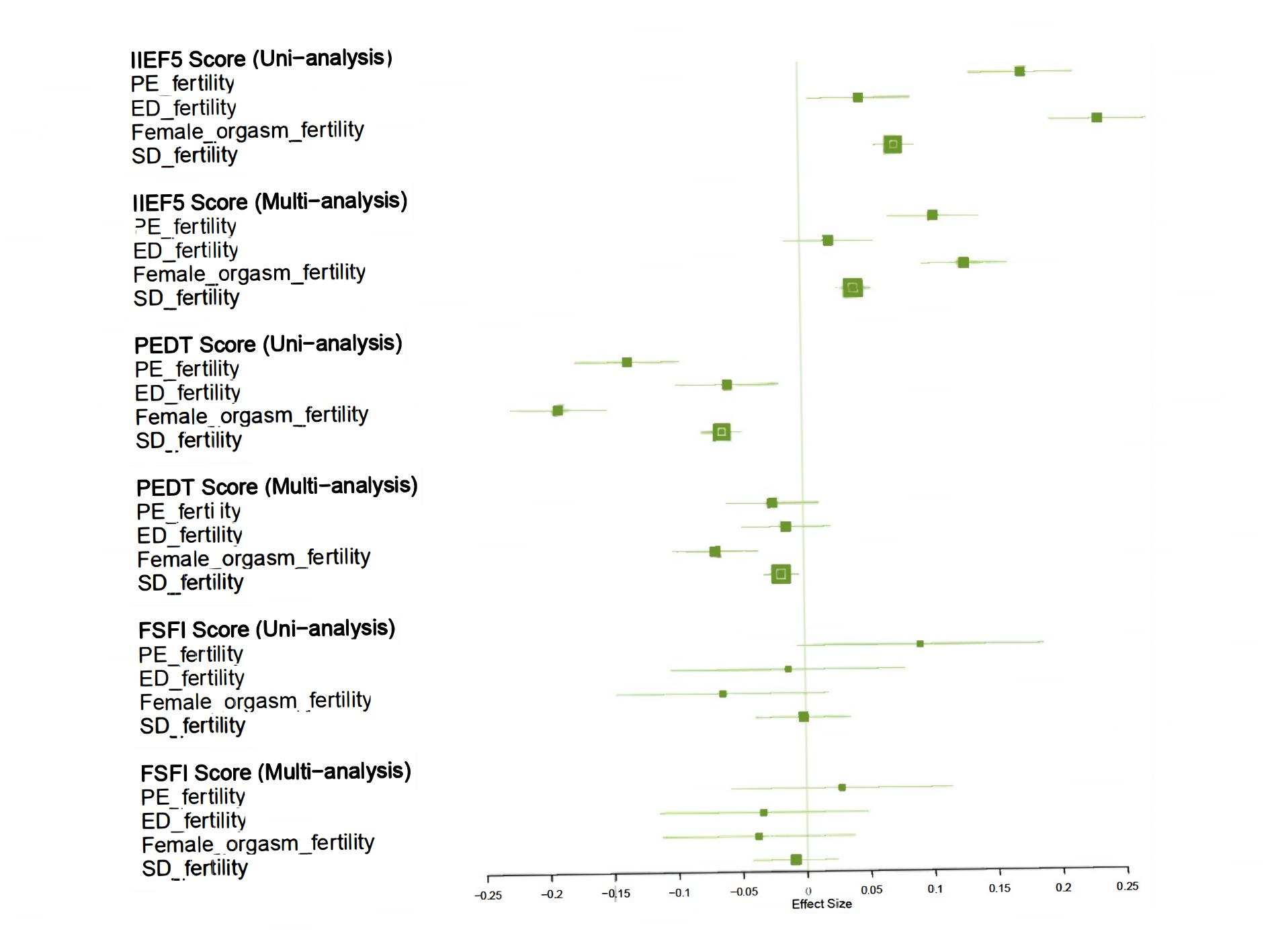


**Figure S1: Effect Sizes for Sexual Function and Fertility Associations in Uni- and Multivariate Analyses**

The figure illustrates the effect sizes of sexual function scores (IIEF-5, PEDT, FSFI-19) on fertility indicators (PE fertility, ED fertility, Female orgasm fertility, SD fertility) in both univariate and multivariate analyses. IIEF-5 and PEDT scores represent man sexual function, while FSFI-19 scores represent women sexual function. Effect size is used to measure the strength of the statistical association, with positive values indicating a positive correlation and negative values indicating a negative correlation. Univariate analysis (Uni-analysis) and multivariate analysis (Multi-analysis) consider different numbers of variables influencing the outcomes.
